# Production of Novel Thermostable Esterases from *Thermus thermophilus* Strain ET-1 in *Escherichia coli* and *Thermus thermophilus* HB27 Using the Bifunctional Expression System pTGT-1 and Characterization of the Recombinant Enzymes

**DOI:** 10.3390/ijms27031372

**Published:** 2026-01-29

**Authors:** Bernardita Valenzuela, Mayra Cayo, Francisco Solís-Cornejo, María-Belen Reyes, Ignacia Palma, Elena Uribe, Pedro Zamorano

**Affiliations:** 1 Departamento de Educación, Facultad de Educación, Universidad de Antofagasta, Antofagasta 1240000, Chile; 2 Laboratorio de Microorganismos Extremófilos, Instituto Antofagasta, Universidad de Antofagasta, Antofagasta 1240000, Chile; mayra.cayo@uamail.cl (M.C.); francisco.solis@uamail.cl (F.S.-C.); 3 Departamento Biomédico, Facultad de Ciencias de la Salud, Universidad de Antofagasta, Antofagasta 1240000, Chile; 4 Departamento de Bioquímica y Biología Molecular, Facultad de Ciencias Biológicas, Universidad de Concepción, Concepción 4070386, Chile; marireyesc@udec.cl (M.-B.R.); igpalma2021@udec.cl (I.P.); auribe@udec.cl (E.U.)

**Keywords:** *Thermus*, lipases, esterases, thermozymes

## Abstract

The thermophilic bacterium *Thermus thermophilus* represents a crucial genetic reservoir for exploring thermostable enzymes as valuable biocatalysts for industrial and biotechnology applications. Here, we identify, clone, and characterize Ces1-ET, Est1-ET, and Plp1-ET, three lipolytic enzymes obtained from *T. thermophilus* strain ET-1 isolated from El Tatio Geothermal Field in Northern Chile. To enable recombinant expression, we constructed the pTGT-1 expression system, a versatile bifunctional shuttle vector compatible with both *Escherichia coli* and *T. thermophilus*. The three thermoenzymes Ces1-ET, Est1-ET, and Plp1-ET, were successfully cloned, expressed, and purified using the pTGT-1 system, with a molecular mass of 25 kDa, 36 kDa, and 28 kDa, respectively. The recombinant purified enzymes displayed optimal temperatures at 60 °C, 80 °C, and 70 °C and optimal pH of 7.5, 9.0, and 8.0 for Ces1-ET, Est1-ET, and Plp1-ET, respectively. Functional biochemical assays revealed a broad tolerance to surfactants, detergents, divalent cations, and high salinity, relevant properties for their application in an industrial setting. These thermostable esterases expand the repertoire of thermozymes from *Thermus* spp., introducing pTGT-1 as an innovative tool for thermophilic protein expression and highlighting *T. thermophilus* strain ET-1 from El Tatio Geothermal Field as a valuable source of thermostable enzymes for industrial and biotechnology applications.

## 1. Introduction

Thermostable esterases represent an important group of biocatalysts due to their ability to function in harsh physicochemical conditions, mainly high temperature, extreme pH and in the presence of surfactants or high salinity [1,2]. These enzymes hydrolyze ester bonds (ester, carbonyl ester, phosphoric ester, and sulfuric ester), generating alcohols and carboxylic acid. However, under certain conditions, they may also catalyze transesterification reactions, positioning them as versatile tools for industrial biotransformations [3,4]. True esterases, commonly referred to as carboxylesterase (EC 3.1.1.1), are serine-dependent enzymes with high substrate specificity that act mainly on short-chain acyl esters [5,6]; in contrast, phospholipases (EC 3.1.1.32 and EC 3.1.1.4) specifically catalyze phospholipid hydrolysis.

Thermophilic microorganisms are a rich reservoir of robust enzymes, particularly lipolytic thermozymes, adapted to high temperature, high ionic strength, and chemical stressors, offering some advantages over enzymes from mesophilic microorganisms [1]. Among thermophiles, *T. thermophilus* is a valuable chassis for thermozymes expression due to its genetic stability, natural transformations, and the development of molecular tools, such as bifunctional vectors, adaptable for expression in both mesophilic and thermophilic microorganisms [7,8,9]. Only a third of thermophilic genes can be expressed as functional proteins in *E. coli* [7,8,10], and this lack of functionality is mainly due to misfolding or the expression of inactive enzymes. Successful expression of enzymes has been demonstrated in the thermophilic bacterium *T. thermophilus*, achieving overexpression levels comparable to those in *E. coli*, underscoring the importance of this microorganism and the development of shuttle vectors.

Thermostable esterases have gained particular interest due to their relevance in industrial biotechnology and their potential contribution to the circular economy. Their stability at high temperatures allows their use in thermal environments with low risk of contamination with mesophiles. Furthermore, high temperatures increase enzyme reaction rates and substrate solubility, making them suitable for thermophilic fermentations, biodiesel synthesis, and polymer degradation, including aliphatic polyesters and PET-related intermediates, where elevated temperatures enhance polymer dynamics and hydrolysis efficiency [11,12].

Recently, we isolated *T. thermophilus* strain ET-1 from El Tatio Geothermal Field in Northern Chile, a poly-extreme habitat characterized by high temperatures (80 °C), high solar radiation and acidic and alkaline fumaroles [13]. Strain ET-1 shows a growth profile with an optimum of 80 °C and pH 8, suggesting that this strain may encode novel thermozymes with biotechnological potential. In this study, we identify, clone, and characterize three esterases (Ces1-ET, Est1-ET, and Plp1-ET) from the draft genome of ET-1, using a newly constructed bifunctional vector, functional in *E. coli* and *T. thermophilus*. We report the biochemical and kinetic properties of these recombinant enzymes and evaluate their performance under industrially relevant conditions, including high temperature, alkaline pH, salt, surfactants, and commercial detergents. These findings expand the catalog of thermostable esterases from *Thermus* spp. and highlight the biotechnological value of extremophiles from the Andean geothermal ecosystems [14].

## 2. Results

### 2.1. Identification of Esterases Genes

**Figure 1:**
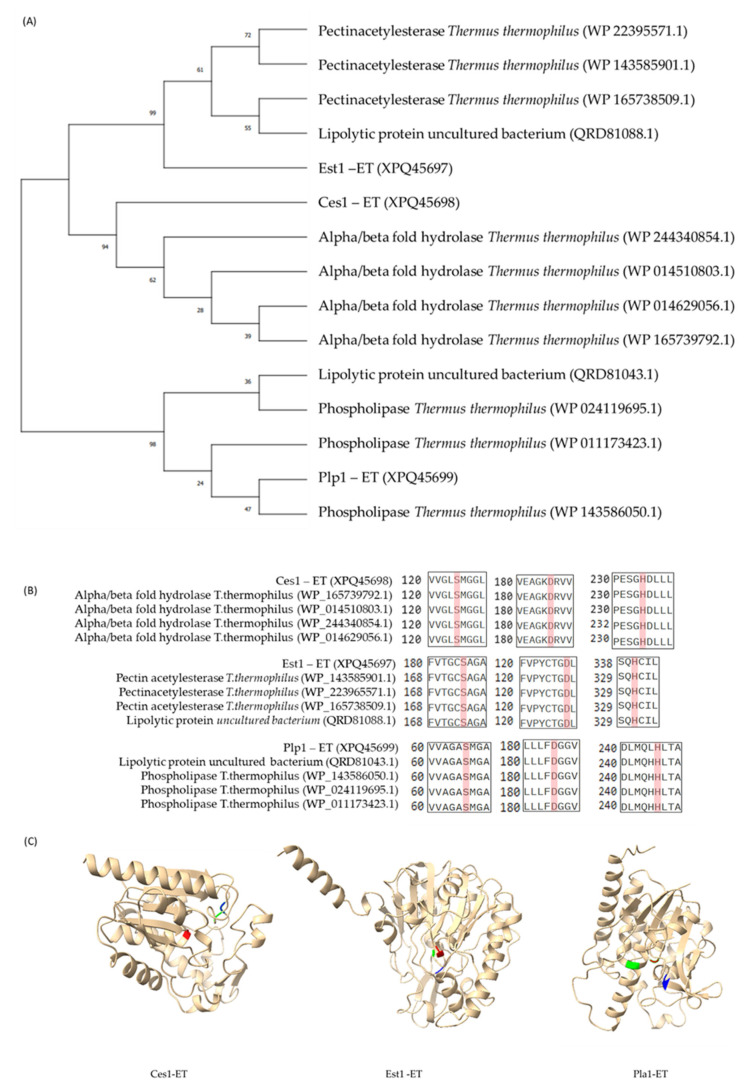
Molecular characterization of the studied esterases. (**A**) Phylogenetic analysis of Ces1-ET, Est1-ET and Plp1-ET sequences. (**B**) Amino acid compositions of the residues forming the catalytic triad of thermophilic esterases; in red is highlighted the catalytic residues. (**C**) Structural predictions generated with AlphaFold and visualized in ChimeraX. The catalytic residues are displayed in different colors: serine in red, aspartic acid in blue, and histidine in green. Complete sequence alignments for each enzyme sequence are provided in Figure A1, Figure A2 and Figure A3 (Appendix A).

**Figure A1:**
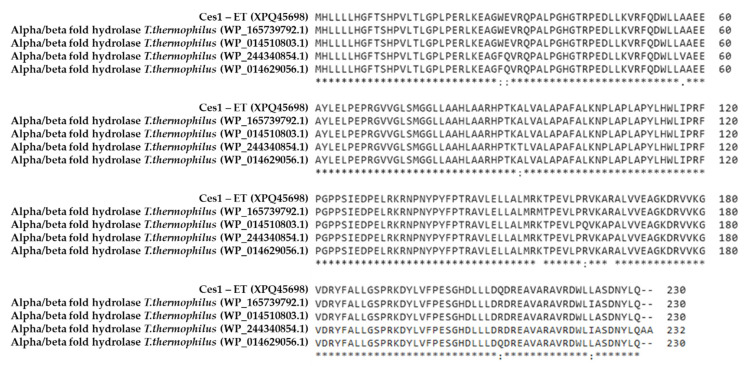
Alignment of Ces1-ET with 4 homologous sequences available in GenBank, where “*” explains total identity, “:” conserved substitution, and “ “ a non-conserved amino acid.

**Figure A2:**
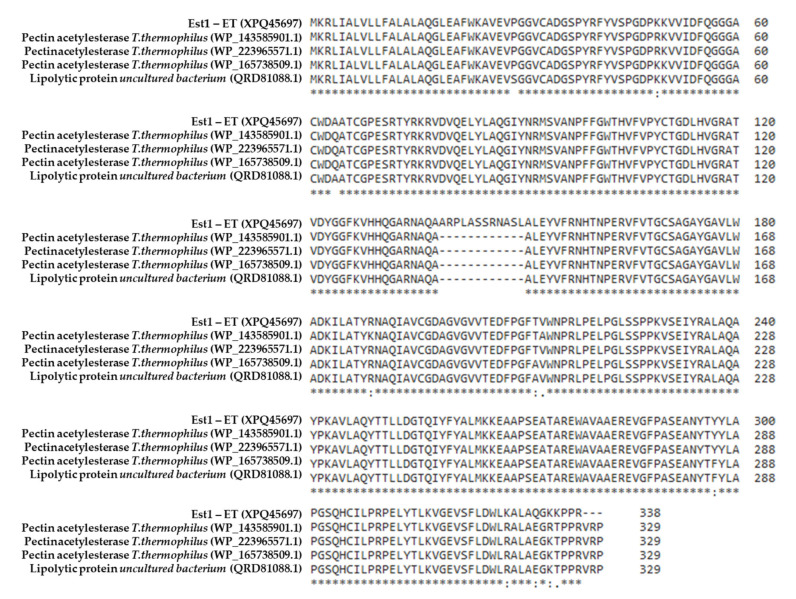
Alignment of Est1-ET with 4 homologous sequences available in GenBank, where “*” explains total identity, “:” conserved substitution, and “ “ a non-conserved amino acid.

**Figure A3:**
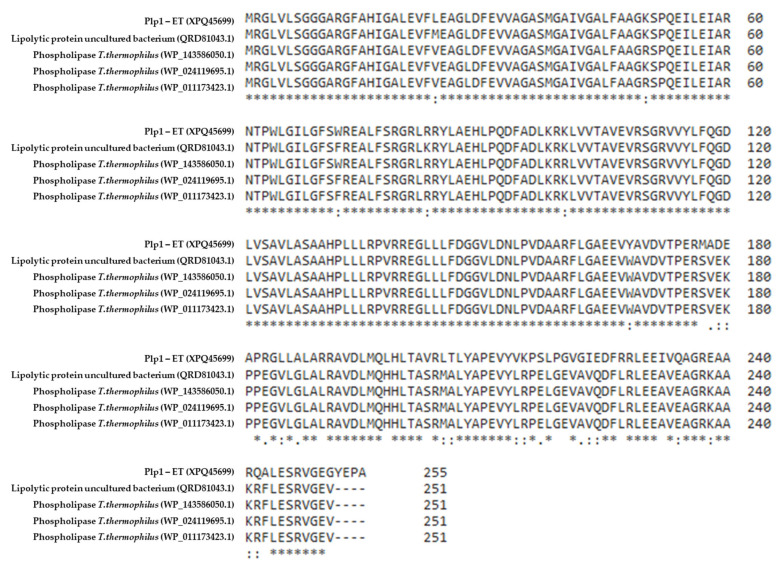
Alignment of Plp1-ET with 4 homologous sequences available in GenBank, where “*” explains total identity, “:” conserved substitution, and “ “ a non-conserved amino acid.

Based on the preliminary draft of partial genome sequencing for strain ET-1 (accession number NCBI BioProject: PRJNA1134705), bioinformatic analysis revealed the presence of three putative genes encoding lipolytic enzymes, namely *Ces1-ET*, *Est1-ET*, and *Plp1-ET*. Blast analysis within the National Center for Biotechnology Information (NCBI) Database (https://www.ncbi.nlm.nih.gov/refseq/; accessed on 11 September 2023) [9] identified proteins with similar amino acid and nucleotide sequences. *Ces1-ET* shows a 97.8% identity with the amino acid sequence of an alpha/beta fold hydrolase from *T. thermophilus* (WP_014629056.1) and a 98.4% similarity with the nucleotide sequences from two enzymes: monoacylglycerol lipase from *T. thermophilus* TTHBAR1 (LR027517.1) and esterase/lipase from *T. thermophilus* JL-18 (CP003252.1). *Est1-ET* shares a 94.9% identity with the amino acid sequences of a pectin acetylesterase from *T. thermophilus* (WP_223965571.1) and a 98.0% similarity with the nucleotide sequences of a hypothetical protein from *T. thermophilus* AK1-1 (AP024937.1). As for *Plp1-ET*, it exhibits an 87.2% identity with the amino acid sequence of a patatin-like phospholipase from *T. thermophilus* (WP_143586050.1) and a 90.2% similarity with the nucleotide sequences of a patatin-like phospholipase from *T. thermophilus* HB27 (CP053287.1). The three genes’ closest sequences in both amino acids and nucleotides are presented in Table 1. A phylogenetic tree was constructed to classify these genes within the lipolytic enzyme family IV (Figure 1A). Additionally, a comprehensive multiple sequence alignment uncovered a characteristic catalytic triad (Ser-Asp-His) (Figure 1B), further supporting the lipolytic nature of these enzymes. Predicted molecular structures generated using AlphaFold display this catalytic triad (Figure 1C). Alignments for each enzyme with four homologous sequences available in GenBank are also shown in Figure A1, Figure A2 and Figure A3; Appendix A.

These analyses allow the classification of these gene sequences from *T. thermophilus* Strain ET1 as a carboxylesterase (*Ces1-ET*), an esterase (*Est1-ET*), and a phospholipase (*Plp1-ET*). The amino acid sequences for these enzymes are shown in Table A1, Table A2 and Table A3; Appendix A.

### 2.2. PCR and Cloning

Specific primers were designed based on the gene sequences (see Table 2) to amplify the open reading frames (ORFs) of the different esterases. Using genomic DNA from *T. thermophilus* strain ET-1, three genes were successfully amplified: 705 bp (*Ces1-ET*), 987 bp (*Est1-ET*) and 771 bp (*Plp1-ET*). The amplicons were cloned into the pGEM-T easy vector (Promega, Madison, WI, USA).

**Figure 2:**
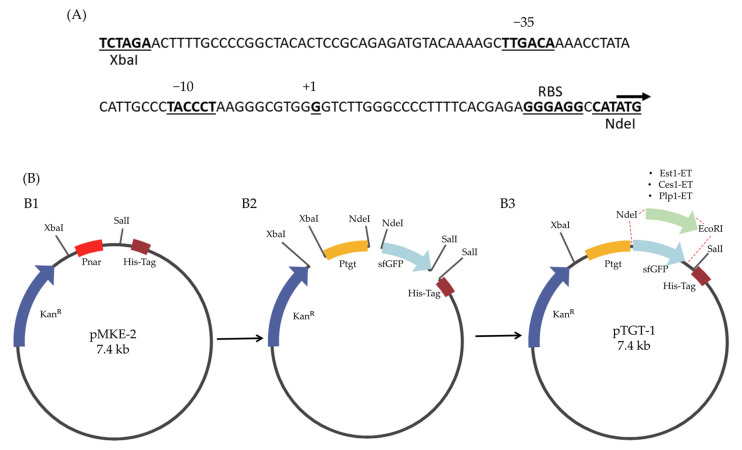
Construction of the pTGT-1 shuttle vector and subcloning strategy for thermophilic esterases. (**A**) Sequences of the Ptgt-promoter indicating the −35 and −10 regions, the transcript start site (+1), and the ribosome binding site (RBS); arrows indicate translation orientation. (**B**) Construction of pTGT-1 from pMKE-2 plasmid. (**B1**) Replacement of Pnar promoter with Ptgt promoter using XbaI and SalI sites. (**B2**) Inserting of Ptgt and the sfGFP ORF via the NdeI and SalI sites. The sfGFP ORF was replaced for esterase RFs (Ces1-ET, Est1-ET and Plp1-ET) using the NdeI and EcoRI sites (**B3**).

**Figure A4:**
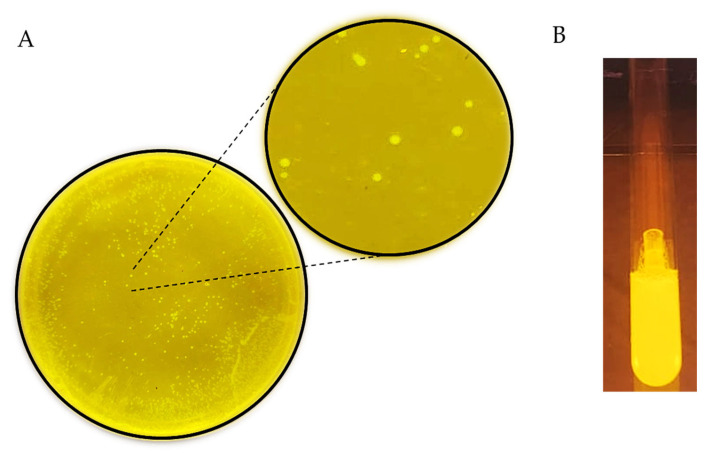
Recombinant expression of superfold GFP (sfGFP) in *Thermus thermophilus* transformed with pTGT-1-sfGFP. (**A**) Culture plate with visibly fluorescent CFUs excited with blue light (480 nm). (**B**) Microbiological liquid culture excited with blue light (480 nm).

The pTGT-1 vector was engineered from the pMKE-2 vector by replacing the Ptgt-promoter and inserting the sfGFP ORF as a reporter (Figure 2). The resulting plasmid, designated pTGT-1, demonstrated functionality by enabling recombinant expression of sfGFP at temperatures up to 50 °C in *T. thermophilus* (Figure A4; Appendix A).

**Figure A5:**
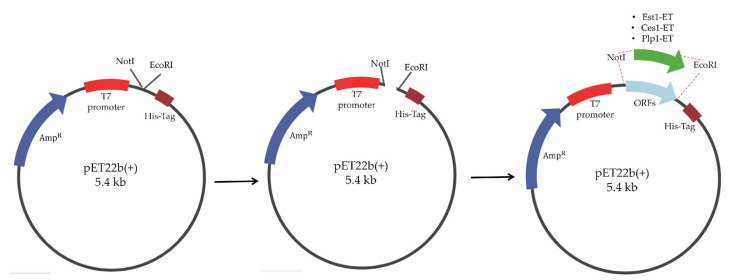
Subcloning schemes of Ces1-ET, Et1-ET and Plp1-ET in the pET22b vector.

The esterases’ ORFs were subsequently cloned in the pTGT-1 plasmid and into the commercial expression vector pET22b(+) (Figure A5; Appendix A).

### 2.3. Recombinant Expression and Purification of Esterases

**Figure 3:**
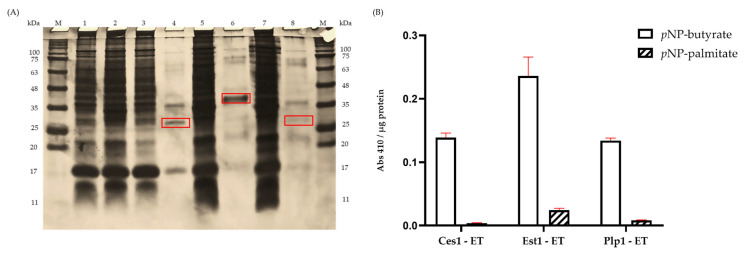
Characterization of recombinant esterases expressed in *E. coli*. (**A**) SDS-PAGE analysis of heterologous expression and purification of esterases in *E. coli*. M, molecular weight markers; lane 1, crude extract of *E. coli* (TOP10); lane 2, empty vector control (pTGT1-GFP); lanes 3 and 4, partially purified and crude extracts of Ces1-ET; lanes 5 and 6, Est1-ET; lanes 7 and 8, Plp1-ET. Enzyme sizes are highlighted in red. (**B**) Substrate specificity assay. Relative activities of Ces1-ET, Est1-ET, and Plp1-ET toward *p*-nitrophenyl-butyrate and *p*-nitrophenyl-palmitate, considering the Abs 410 nm/µg protein. Data are presented as mean ± SD (*n* = 3), and the red line in the image indicates the standard deviation when performing the experiments.

**Figure A6:**
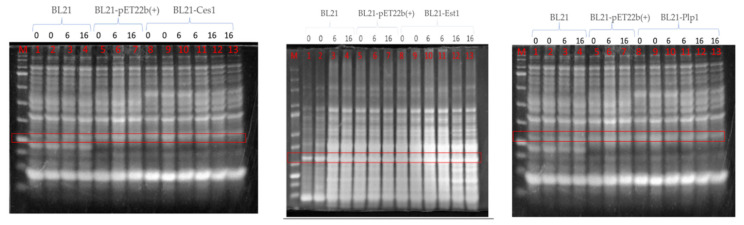
SDS-PAGE of the crude extract of recombinant expression of Ces1-ET, Et1-ET and Plp1-ET/pET22b vectors in *Escherichia coli* BL21 (DE3). The red numbers indicate the lanes of the SDS-PAGE gels for the three esterases, analyzed at different IPTG induction times. Lanes 1–4 correspond to the BL21 (DE3) host without plasmid; lanes 5–7 correspond to the BL21 (DE3) host carrying the empty pET-22b plasmid; and lanes 8–13 correspond to the host carrying the pET-22b plasmid containing the esterase ORF (Ces1, Est1, and Plp1, respectively). The red box indicates the expected molecular size of the overexpressed recombinant protein.

Recombinant esterases were constitutively expressed after 16 h of growth in *E. coli* (TOP10) and *T. thermophilus* HB27 using the pTGT-1 expression plasmid. The esterases were purified from total cellular lysates through affinity chromatography on a nickel nitrilotriacetate column, and the fractions were analyzed by SDS-PAGE gels (Figure 3A). Bands of the expected molecular masses were observed: 25 kDa for Ces1-ET, 36 kDa for Est1-ET, and 28 kDa for Plp1-ET. No detectable expression was observed using the pET22b(+) vector in *E. coli* (TOP10). SDS-PAGE gels are shown in Figure A6; Appendix A.

### 2.4. Esterase Activity

To determine the optimal substrate for the enzyme activity assay, two *p*-nitrophenyl esters with acyl chains of varying lengths, butyrate (C4) and palmitate (C16), were used. Hydrolysis of the ester bond is visualized by the release of p-nitrophenol, indicated by a yellow color, and the absorbance was measured at 410 nm. In the lipolytic assay, *p*-nitrophenyl-butyrate (*p*NP-butyrate) proved to be the most efficient substrate for all three enzymes, while a low esterase activity was exhibited towards the *p*-nitrophenyl-palmitate substrate (Figure 3B).

For the biochemical characterization of the purified enzymes and crude extracts, *p*NP-butyrate was used as the standard substrate.

### 2.5. Temperature Effect on Esterase Activity

**Figure 4:**
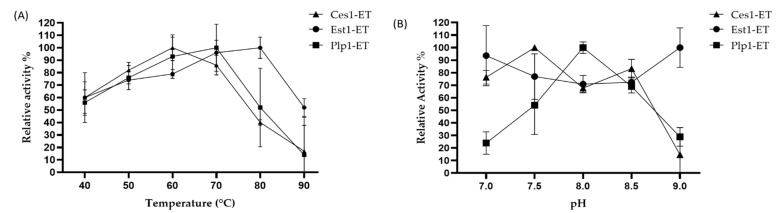
Properties of Ces1-ET, Est1-ET, and Plp1-ET expressed in *E. coli*. (**A**) Effect of temperature on enzymatic activity in the range of 40–90 °C. (**B**) Effect of pH on enzyme activity using 50 mM Tris-HCl buffer across pH of 7.0–9.0. The extinction coefficient of *p*-nitrophenol was determined at various pH values using a 25 mM solution. The resulting values (in M^−1^·cm^−1^) were: pH 7.0–11.0, 360; pH 7.5–15.0, 400; pH 8.0–15.0, 400; pH 8.5–18.0, 120; pH 9.0–20.0, 080. These coefficients were subsequently used to correct the relative enzymatic activity measurements across pH conditions. Enzymatic activity for optimal temperature was assessed by incubating the enzyme for 60 min at the indicated temperature. Assays for optimal pH were performed by incubating for 60 min at each enzyme’s optimal temperature and the corresponding pH. Similar determinations were carried out using purified enzymes expressed in *T. thermophilus* (Figure A7).

The temperature profile of Ces1-ET activity was maintained above 50% between 40 and 70 °C, with an optimum at 60 °C, but retained less than 20% activity at 90 °C. Est1-ET exhibited more than 70% activity between 60 and 80 °C, with optimal activity at 80 °C. At 90 °C, Est1-ET retained approximately 50% activity. Plp1-ET showed catalytic activity between 40 and 90 °C, with an optimum at 70 °C, and retained nearly 50% activity at 80 °C and less than 20% at 90 °C (Figure 4A).

### 2.6. pH Effect on Esterase Activity

The pH dependency analysis of Ces1-ET exhibited more than 50% relative activity at pH between 7.0 and 8.5, reaching its optimal activity at approximately pH 7.5, Est1-ET revealed that the enzyme displays more than 50% relative activity within the pH range 7.0–9.0, with a maximum activity at pH 9.0. In contrast, Plp1-ET showed more than 50% relative activity between pH 7.5 and 8.5, with its highest activity at pH 8.0 (Figure 4B).

### 2.7. Kinetic Parameters of Enzymatic Activity

**Figure A8:**
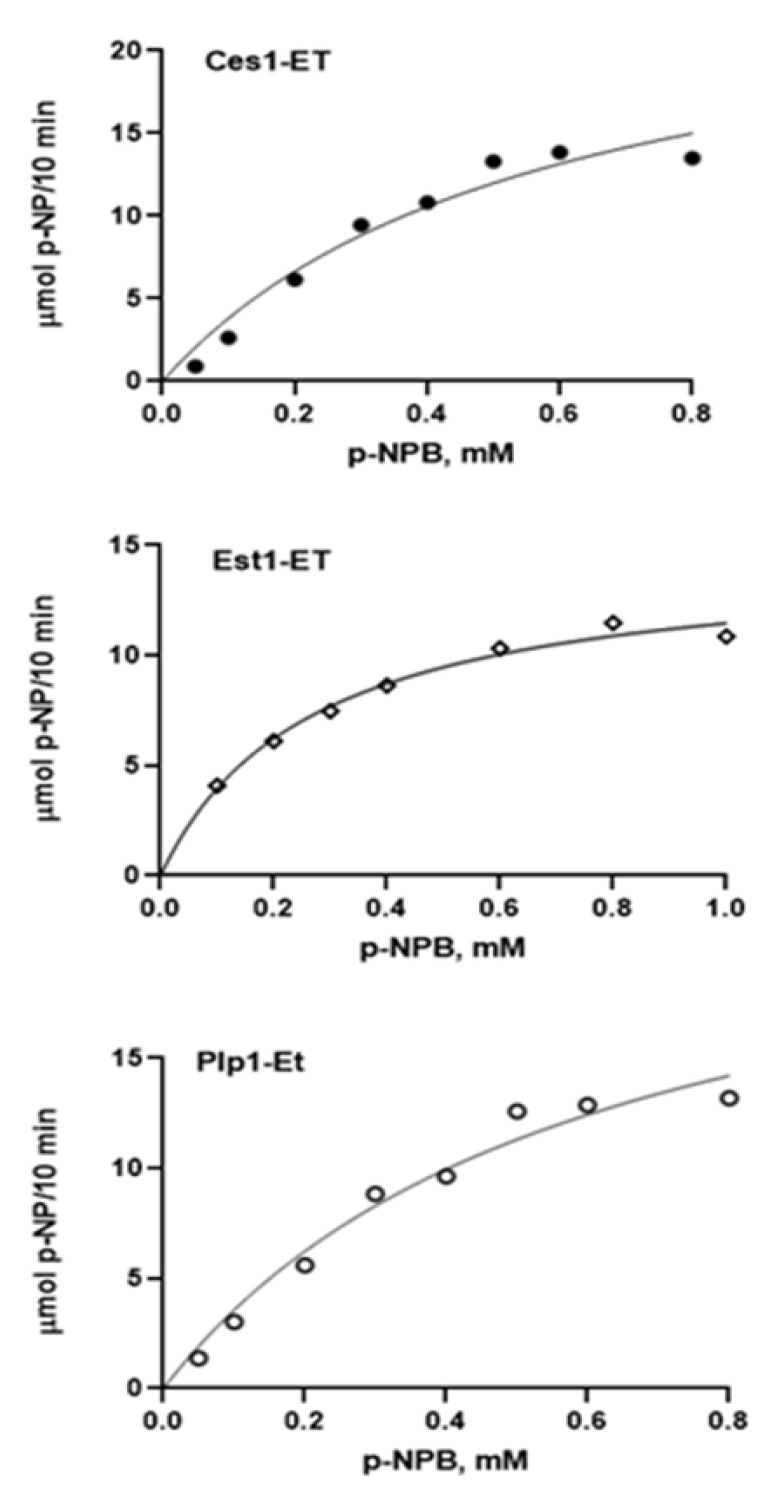
Michaelis–Menten plot for the esterase activity of Ces1-ET, Est1-ET and Plp1-ET. Activity was performed at 55 °C and 50 mM Tris-HCl at pH 8.0 using *p*NP-butyrate as the substrate. At each substrate concentration, the spontaneous hydrolysis of *p*NP-butyrate was subtracted. The graph shows a representative result from three independent experiments.

**Figure A9:**
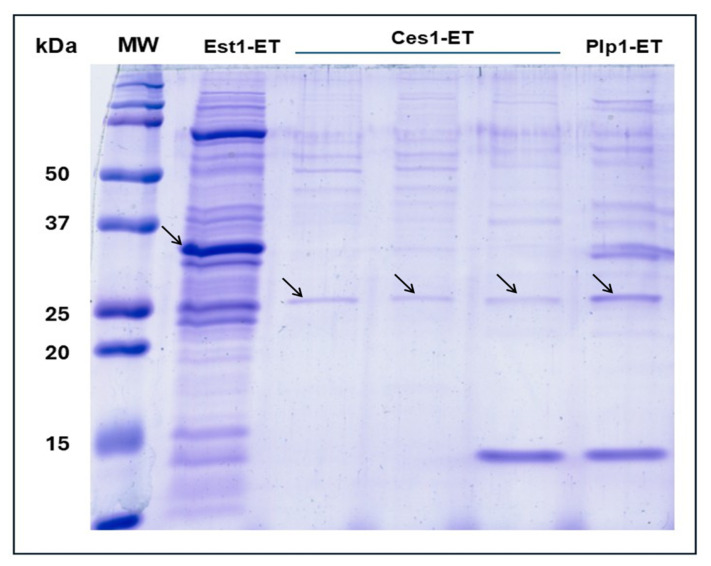
SDS-PAGE partially purified by the mean of NTA-Ni^2+^ affinity chromatography in the AKTA start protein purification system. The arrows indicate the size of the enzymes.

The kinetic properties of the recombinant esterases Ces1-ET, Est1-ET, and Plp1-ET, as presented in Table 3, reveal minor differences in their catalytic efficiencies and substrate affinities. Ces1-ET exhibits a maximum reaction velocity (*Vmax*) of 15 ± 4 μmoles *p*-nitrophenol/10 min/1 µg protein. In contrast, Est1-ET exhibits a maximum reaction velocity (*Vmax*) of 13 ± 2 μmoles *p*-nitrophenol/10 min/1 µg protein, indicating a relatively lower catalytic activity compared to the other two enzymes. Plp1-ET shows a *Vmax* value of 18 ± 4 μmoles *p*-nitrophenol/10 min/1 µg protein. Regarding substrate affinity, Ces1-ET has a Michaelis constant (*Km*) of 0.25 ± 0.05 mM, and Est1-ET presents a *Km* of 0.20 ± 0.01 mM, while Plp1-ET presents a *Km* of 0.30 ± 0.05 mM, suggesting a similar affinity to the substrate by the enzymes. Michaelis–Menten curves for Ces1-ET, Est1-ET and Plp1-ET were generated at 55 °C and pH 8.0, using *p*NP-butyrate as the substrate (Figure A8 and Figure A9).

### 2.8. Effect of Divalent Cations on Esterase Activity

The effect of divalent cations at 2 mM and 10 mM is shown in Table 4. In the presence of 2 mM CaCl_2_, the relative enzymatic activity of Ces1-ET and Plp1-ET remains largely unchanged compared to the control; however, Est1-ET exhibits a 35% increase in activity. When the concentration of this ion is increased to 10 mM, activity decreases by 10% for Ces1-ET and Plp1-ET, whereas Est1-ET shows a slight increase of 10%.

In the presence of 2 mM MgCl_2_, only minor variations were observed for the three esterases. At 10 mM, activity decreased by 24% for Ces1-ET, 7% for Est1-ET, and 21% for Plp1-ET.

In the presence of MgSO_4_, a slight variation was observed at 2 mM, whereas, at 10 mM, a decrease of 17%, 41% and 27% was observed for Ces1-ET, Est1-ET and Plp1-ET, respectively.

In the presence of 2mM ZnSO_4_, all enzymes showed an increase in relative activity, with Ces1-ET increasing by 8%, Est1-ET by 38%, and Plp1-ET by 18%. Conversely, at 10 mM, the relative activity decreased by 20% for Ces1-ET, 9% for Est1-ET, and 4% for Plp1-ET.

In the case of CuSO_4_, the relative enzymatic activity decreased at both concentrations. At 2 mM, activity decreased by 20%, 23%, and 10% for Ces1-ET, Est1-ET, and Plp1-ET, respectively. At 10 mM, the decreases became more pronounced, reaching 35%, 55%, and 27% for these enzymes.

Finally, in the presence of 2 mM BaCl_2_, activity increased by 11%, 34%, and 20% for Ces1-ET, Est1-ET, and Plp1-ET, respectively. At 10 mM, however, the relative activity decreased by 27%, 39%, and 41%, respectively.

### 2.9. Effect of Inhibitors on Esterase Activity

The effect of the four enzymatic inhibitors on esterase activity is shown in Table 4. In the presence of β-mercaptoethanol, enzymatic activity decreased at both concentrations. At 1 mM, activity was reduced by 65%, 31%, and 45% for Cest1-ET, Est1-ET, and Plp1-ET, respectively. Notably, no activity was observed at 10 mM.

For PMSF and DTT, inhibition of activity was complete in all three recombinant enzymes at both concentrations tested. At 1 mM EDTA, enzymatic activity decreased by 78%, 51%, and 70% for Ces1-ET, Est1, and Plp1-ET, respectively. At 10 mM of EDTA, activity decreased by 65%, 21%, and 60% for Ces1-ET, Est1-ET, and Plp1-ET, respectively.

### 2.10. Effect of Surfactants on Esterase Activity

The effect of surfactants on esterase activity is shown in Table 4. Overall, most surfactants caused a reduction in enzymatic activity; however, Est1-ET was an exception. This enzyme exhibited a 42% and 34% increase in activity in the presence of Tween 80 at 1% and 10% (*v*/*v*), respectively. A similar trend was observed with Triton X-100, where activity increased by 65% and 66% at 1% and 10%, respectively, compared to the control without surfactants.

In contrast, 1% (*w*/*v*) SDS led to decreased activity in all enzymes, which retained 84%, 32%, and 82% relative activity for Ces1-ET, Est1-ET, and Plp1-ET, respectively. In the presence of 10% (*w*/*v*) SDS, activity dropped significantly, with enzymes retaining only 21%, 14%, and 21%, respectively. On the other hand, both concentrations of LDS caused a severe inhibitory effect, with Est1-ET showing the highest residual activity (26% at 1%). This stronger inhibitory effect of LDS compared to SDS can be attributed to lithium ion, which destabilizes the protein structure more efficiently than sodium, leading to enhanced denaturation and a sharper reduction in enzymatic activity even at lower concentrations. Lastly, CTAB 1% (*w*/*v*) relative activity decreased by 36%, 71%, and 18% for Ces1-ET, Est1-ET, and Plp1-ET, respectively. At 10% (*w*/*v*) CTAB, activity was further reduced, with enzymes retaining only 18%, 17%, and 21% for Ces1-ET, Est1-ET, and Plp1-ET, respectively.

### 2.11. Enzymatic Activity in Commercial Detergent

The effect of liquid commercial detergents on esterase activity is shown in Table 4. Interestingly, for Ces1-ET at a detergent concentration of 1% (*v*/*v*), activity increased 81% in Ariel and 52% in OMO, whereas only slight increases of 1% and 3% were observed in Sun and Quix, respectively. In contrast, in the presence of 1% (*v*/*v*) Perla detergent, activity decreased by 55%. When the detergent concentration increased to 10% (*v*/*v*), activity was completely abolished in Ariel and Quix, while, in OMO, Sun and Perla, activity decreased by 58%, 13% and 65%, respectively.

For Est1-ET, at 1% (*v*/*v*) detergent concentration, activity increased by 2% in Perla, 30% in Quix, and 23% in Sun, whereas, in OMO and Ariel, the activity decreased by 19% and 11%, respectively. When the detergent concentration was increased to 10%, activity decreased in all tested detergents, with relative activity values of 27% (Perla), 26% (OMO), 21% (Ariel), 16% (Quix), and 15% (Sun).

For Plp1-ET, at 1% detergent concentration, activity increased by 30% in OMO and 50% in Ariel while decreasing by 59% in Perla, 10% in Quix, and 20% in Sun. When the detergent concentration increased to 10%, activity decreased in all detergents, retaining a relative activity of 34% (Perla), 40% (OMO), and 50% (Sun), whereas, in Ariel and Quix, activity was completely abolished.

### 2.12. Effect of NaCl on Esterase Activity

The effect of NaCl concentration on enzymatic activity is shown in Table 5. As the NaCl concentration increases, the three recombinant esterases exhibit a progressive decrease in relative activity. In the case of Ces1-ET, activity is maintained at 55% at 2 M of NaCl, dropping to 39% at 4 M of NaCl. For Est1-ET, enzymatic activity remains at 52% at 2 M of NaCl and decreases to 41% at 4 M of NaCl. Similarly, Plp1-ET retains 60% activity at 2 M and 45% at 4 M of NaCl.

## 3. Discussion

The genome-guided identification of three lipolytic enzymes in *T. thermophilus* ET-1 expands the diversity of esterases known for this thermophilic genus [13]. BLAST analysis showed a high identity among similar enzymes found in *Thermus* spp., such as esterases [15], carboxylesterases and patatin-like phospholipases [16,17,18], suggesting a conserved function across geographically distant isolates, which is relevant due to the broad cosmopolitan distribution of *T. thermophilus* [19].

Furthermore, the molecular analyses, including sequence alignments and AlphaFold structure predictions, confirmed the lipolytic nature of Ces1-ET, Est1-ET, and Plp1-ET. The esterases contain the classical Ser-Asp-His catalytic triad, which is essential for enzyme stability and functionality of the enzyme active site [20,21,22]. This triad is considered crucial for the activity and stability of thermostable lipases and esterases from extreme environments, such as hot springs [23,24]. These conserved residues, identified through structural predictions and alignments, underscore a broad evolutionary conservation in thermophilic and alkaliphilic lipases [25,26]. Phylogenetic analysis placed these genes in the lipolytic enzyme family IV [23,27].

Expression of thermoenzymes in mesophilic microorganisms like *E. coli* often results in improper folding, formation of inclusion bodies, and loss of activity [10,28]. Expression of the ORFs of Ces1-ET, Est1-ET, and Plp1-ET into the pET22b(+) system in *E. coli* was unsuccessful, confirming this limitation. Similarly, the lack of expression of thermozymes using the pET22b(+) system has been previously reported [29]. Our engineered bifunctional pTGT-1 plasmid overcomes these limitations, allowing constitutive expression of these esterases in both *E. coli* and *T. thermophilus*. This system integrates the Ptgt promoter derived from the *CarH* gene. Unlike the pMKE-2 vector cassette promoter, which is inducible by microaerophilia and 40 mM potassium nitrate [8], the Ptgt promoter supports consistent protein expression under normal culture conditions without overexpression. This system provides a comparative advantage for evaluating the expression of thermozymes in mesophilic and thermophilic hosts and could be useful in biotechnology pipelines for screening and characterizing thermozymes [30,31].

The biochemical characterization of Ces1-ET, Est1-ET, and Plp1-ET revealed features important for biotechnology and industrial applications. All enzymes demonstrated significant activity at high temperatures, with optimal activity between 60 °C and 80 °C, suggesting their potential for a wide range of temperature applications [32]. Their ability to maintain activity at high temperatures is advantageous for bioprocesses in high-temperature reactors, enzymatic cleaning, biodiesel production or hydrolysis of recalcitrant substrates, where increased thermal energy improves solubility [33,34]. Furthermore, they exhibited significant alkaline activity (pH 8.0–8.5), suggesting a potential application in detergents or waste treatment where high pH is common [35,36,37].

In this study, the *Vmax* and *Km* kinetic parameters of Ces1-ET, Est1-ET, and Plp1-ET were determined using *p*NP-butyrate as a substrate. Plp1-ET exhibited a slightly higher *Vmax*; however, all the enzymes showed comparable efficiencies and *Km* values, indicating similar substrate-binding affinities, which is typical for these structurally related enzymes [4].

Ces1-ET, Est1-ET, and Plp1-ET displayed differential tolerance to ions, surfactants, and commercial detergents. The capacity of Plp1-ET and Ces1-ET to retain activity in the presence of 1–2% detergent, together with Est1-ET’s activation by Tween 80 and Triton X-100, suggests potential application as enzymatic additives in cleaning products, textile processing, or lipid-rich industrial effluents where surfactants are unavoidable. High tolerance to divalent cations, especially Ca^2+^ and Zn^2+^, is advantageous, because calcium-stabilized esterases exhibit improved rigidity and therefore enhanced operational stability in bioprocessing environments [38].

Furthermore, the effects of surfactants and detergents on esterase activity revealed varied responses, including unexpected increases in activity for some enzymes. This finding could support the development of detergent formulations that rely on enzymatic activity for enhanced effectiveness [35,36,37].

From a circular bioeconomy perspective, thermostable esterases are increasingly valuable for the depolymerization of synthetic and natural polyesters, lipid-rich waste valorization, and bioremediation in high-temperature settings [39,40,41]. Thermophilic hydrolysis can improve polymer chain mobility, reduce viscosity, and accelerate depolymerization, advantages particularly relevant for PET-associated cutinase-esterase systems developed for plastic recycling.

Some of the attributes of Ces1-ET, Est1-ET, and Plp1-ET differ from those described for esterases in *T. thermophilus* HB27. Differences in molecular size and optimal pH indicate that these enzymes have unique attributes, exhibiting maximal activity at higher pH and high temperatures [42,43,44,45]. These features may reflect genetic adaptations of *Thermus* populations inhabiting unique Andean thermal ecosystems.

Overall, these results demonstrate that *T. thermophilus* ET-1 is a promising source of novel thermoenzymes and that the pTGT-1 system offers a flexible platform for the production and characterization of thermozymes. The combined thermostability, alkaline tolerance, and detergent compatibility of Ces1-ET, Est1-ET, and Plp1-ET underscore their relevance to industrial biotechnology and sustainable applications in the circular bioeconomy. Future work will focus on protein engineering to enhance catalytic efficiency, as well as evaluating their performance in polyester degradation and thermophilic bioreactor systems.

## 4. Materials and Methods

### 4.1. Identification of Esterases Genes

The partial genome sequencing analysis of *T. thermophilus* ET-1 was conducted using the UniProt UGENE program v49.1 [46]. Three specific genes coding for esterase/lipase enzymes were identified and compared with similar amino acid and nucleotide sequences through BLAST v2.14.0 analysis [47] using the National Center for Biotechnology Information (NCBI) Database (https://www.ncbi.nlm.nih.gov/refseq/; accessed on 11 September 2023). The classification was carried out by examining the enzyme structure using the SMART website [48] (URL: http://smart.embl.de/; accessed on 20 September 2023). Additionally, a comprehensive multiple sequence alignment uncovered a characteristic catalytic triad (Ser-Asp-His), further supporting the lipolytic nature of these enzymes. Meanwhile, prediction of the molecular structures was performed with AlphaFold [49] (URL: https://alphafold.ebi.ac.uk/; accessed on 20 November 2023). To predict the presence of signal peptides in the esterase sequences, SignalP 6.0 software was used.

### 4.2. DNA Extraction, PCR and Cloning

The *Thermus thermophilus* strain ET-1 employed in this study was retrieved from a cryopreserved collection maintained at −80 °C in liquid culture supplemented with 50% glycerol. Subsequent cultivation followed the protocol described by Valenzuela et al. (2024) [14]. Briefly, the strain was initially propagated in liquid ATM medium at 70 °C, after which aliquots were plated on ATM medium solidified with 2% agar. Plates were incubated at 70 °C in a humid chamber for three days, resulting in well-defined isolated colonies suitable for downstream experimentation. Liquid cultures were prepared by inoculating isolated colonies into 10 mL of AMT medium [14] and incubating at 70 °C with constant agitation for 3 days. Following incubation, 6 mL of the culture were harvested and centrifuged at 8000× *g* for 4 min to pellet the bacterial cells. The bacterial pellet was treated with 200 µL of lysis solution (50 mM Tris-HCl, 15 mM NaCl, 10 mM EDTA, and 10% SDS; pH 7.5) supplemented with 30 µL of lysozyme (50 mg/mL) and incubated at 37 °C for 1 h. Subsequently, the lysate underwent freeze–thaw cycles (−80 °C for 5 min, followed by 80 °C for 5 min, repeated twice), was treated with 6 µL of RNAse A (50 mg/mL) at 37 °C for 30 min, and then mixed with 400 µL of 50 mM Tris-HCl (pH 7.5) and 400 µL of phenol:chloroform:alcohol (125:24:1). After centrifugation at 10,000× *g* for 10 min at 4 °C, the aqueous phase was recovered, transferred, and subjected to a second extraction with phenol:chloroform:alcohol, followed by chloroform:alcohol (25:1). The resulting aqueous phase was precipitated with cold absolute ethanol, washed with 70% ethanol, air-dried, and resuspended in 30 µL of nuclease-free water at 42 °C for 10 min.

Genomic DNA from *T. thermophilus* strain ET-1 served as a template for amplifying the esterase genes with specific primers designed for Ces1-ET (Ces1-FP and Ces1-RP), for Est1-ET (Est1-FP and Est1-RP) and for Plp1-ET (Plp1-FP and Plp1-RP) (sequences shown in Table 2). These primers were designed to incorporate NdeI/NotI and EcoRI restriction sites. The polymerase chain reaction (PCR) conditions consisted of an initial denaturation (94 °C, 1 min), followed by 35 cycles of denaturation (94 °C, 30 s), annealing (53 °C, 30 s), extension (72 °C, 2 min), and a final extension step (72 °C, 10 min) using Taq DNA polymerase High Fidelity (New England Biolab, Ipswich, MA, USA). PCR products were cloned into the pGEM-T Easy plasmid (Promega, Madison, WI, USA) and integrated into the pTGT-1 and the commercial pET22b(+), (Novagen, Madison, WI, USA).

### 4.3. Plasmid Construction and Subcloning of Esterases ORFs

The Ptgt promoter sequence (122 bp) was synthesized in silico, incorporating −35 and −10 regions with a transcriptional spacer (17 bp), a ribosome binding site (RBS) sequence, the transcription start site (+1), and XbaI and NdeI restriction sites. The design included a defined translation start (Figure 2A). Simultaneously, the coding sequence (ORF) of super-folded green fluorescent protein sfGFP was chemically synthesized, incorporating NdeI sites at the 5’ end and EcoRV, EcoRI, and SalI sites at the 3’ end. Plasmid assembly for pTGT-1 was performed using XbaI, NdeI, and SalI restriction sites, as depicted in Figure 2B.

The subcloning of the three esterase sequences (Ces1-ET, Est1-ET, and Plp1-ET) into the pTGT-1 vector was carried out using NdeI and EcoRI sites, resulting in the subsequent constructs: pTGT1-Ces1, pTGT1-Est1, and pTGT1-Plp1, as illustrated in Figure 2B. The subcloning of the three esterase sequences (Ces1-ET, Est1-ET, and Plp1-ET) into the pET22b(+) vector was performed using NotI and EcoRI restriction sites, yielding the constructs pET22b-Ces1, pET22b-Est1, and pET22b-Plp1 (Figure A2, Appendix A).

The transformation of *T. thermophilus* HB27 was performed following a modified protocol based on Berenguer [50]. A single colony was cultivated in AMT medium [13] for 14 days. This culture was then used to inoculate a sterile 10 mL tube containing 3 mL of AMT medium, starting with an initial optical density (OD) of 0.1 (550 nm). The culture was incubated at 70 °C with agitation until an OD of 0.3 (550 nm). Subsequently, 1 mL of the cultured broth was transferred to a tube preheated to 70 °C, where 100 ng of pTGT-1 vector was added. The mixture was incubated with agitation at 70 °C for 1 h. After incubation, the culture was centrifuged at 10,000× *g* rpm for 2 min to remove the supernatant, retaining approximately 200 μL for cell resuspension. The resuspended cells were then plated onto preheated Petri dishes containing AMT medium supplemented with kanamycin (50 mg/mL). Plates were incubated in a humid chamber at 55 °C for 2–3 days until visible colonies were formed.

### 4.4. Recombinant Expression and Purification of Esterases

The expression of the esterases was carried out using the pET22b(+) and pTGT-1 vector systems in *E. coli* TOP10 and *T. thermophilus* HB27, respectively. A single colony with the plasmids containing the ORFs of the esterases containing a 6xHis tag was grown at 37 °C or 70 °C during 24 h in a rotary shaker (LabNet 311DS, Edison, NJ, USA) at 1500 rpm in Erlenmeyer flasks loaded with LB broth (pH 7.5) or AMT medium, both supplemented with 50 μg/mL kanamycin. Purification of the esterases was carried out using Protino Ni-NTA agarose (Macherey-Nagel, Düren, Germany). Briefly, the bacterial pellet was homogenized in lysis buffer (50 mM NaH_2_PO_4_, 300 mM NaCl, and 10 mM imidazole, pH 8.0) and loaded into an equilibrated column. The column was washed with wash buffer (50 mM NaH_2_PO_4_, 300 mM NaCl, and 20 mM imidazole, pH 8.0), and the purified protein was eluted in elution buffer (50 mM NaH_2_PO_4_, 300 mM NaCl, and 250 mM imidazole, pH 8.0). The eluted proteins were dialyzed in 50 mM Tris-HCl (pH 8.0) before characterization assays.

Sodium dodecyl sulfate-polyacrylamide gel electrophoresis (SDS-PAGE) was conducted following the Laemmli protocol [51]. A vertical slab mini-gel system (BIO-RAD, Hercules, CA, USA) with a 12% separating gel was used, applying 120 mA for 6 h. Each sample (75 μL) was mixed with 25 μL of loading buffer (BIO-RAD, Hercules, CA, USA), homogenized, incubated at 99 °C for 5 min, and loaded onto the gel. Post-migration, the gels underwent protein detection through staining with silver nitrate. A broad-range SDS-PAGE molecular weight standard, AccuRuler RGB Plus Prestained Protein Ladder (MaestroGen Inc., Hsinchu City, Taiwan), was used as reference.

### 4.5. Substrate Specificity

For the initial tests, a solution was prepared using 50 mM of Tris-HCl buffer, pH 7.5, which contained *p*NP-butyrate or *p*NP-palmitate as a substrate for lipolytic activity. To determine substrate specificity, each reaction contained 5 µL of one of the enzymes (Ces1-ET, Est1-ET and Plp1-ET); 5 µL of 10 mM specific substrate; 20 µL of absolute ethanol and 470 µL of Tris-HCl buffer, pH 7.5. Reactions were incubated at 60 °C for 1 h in a dry incubator (LabNet 311DS), after which absorbance was measured at 410 nm. Blanks without enzymes were included to incorporate any non-enzymatic changes in absorbance. This blank contained all the components of the reaction mixture except the enzymes.

### 4.6. Lipolytic Activity Assay

Lipolytic activity was determined in solution using 10 mM *p*NP-butyrate as the substrate, with product formation monitored spectrophotometrically at 410 nm [52], and in 50 mM Tris-HCl buffer (pH 8.0) at 70 °C over 60 min of reaction time. One activity unit was defined as the amount of enzyme that released 1 μmol of *p*-nitrophenol per minute under standard assay conditions. All measurements were carried out in triplicate.

### 4.7. Temperature Effect on Esterase Activity

The effect of temperature on the esterase activity was evaluated as described in Section 4.6, using the *p*NP-butyrate as a substrate in 50 mM Tris-HCl buffer at pH 8.0 at different temperatures in the range of 40–90 °C. All measurements were carried out in triplicate.

### 4.8. pH Effect on Esterase Activity

The determination of the optimal pH for the recombinant esterases was evaluated similarly as described in Section 4.6, using *p*NP-butyrate as a substrate in 50 mM Tris-HCl buffer at different temperatures (Ces1-ET at 60 °C, Est1 at 80 °C and Plp1-ET at 70 °C). The different pH values were obtained using 50 mM Tris-HCl adjusted pH values 7.0, 7.5, 8.0, 8.5, and 9.0 at the indicated temperatures. The extinction coefficients were determined using a 25 µM solution of *p*-nitrophenol (*p*NP) at each pH value tested. From these measurements, the corresponding extinction coefficients were calculated, and a correction factor was obtained for each pH. These pH-specific correction factors were applied to adjust the absorbance values recorded in the esterase assays used to determine the optimal pH of the enzymes.

### 4.9. Esterases Purification and Kinetic Parameters

Ces1-ET, Est1-ET and Plp1-ET were partially purified by means of NTA-Ni^2+^ affinity chromatography in the AKTA Start Protein Purification System (Cytiva Company, Marlborough, MA, USA). Proteins were eluted with 100–200 mM imidazole and subsequently dialyzed against 50 mM Tris-HCl buffer (pH 8.0) to remove imidazole interference. For determining the kinetic parameters, esterase activity was carried out at 60 °C in a 50 mM Tris-HCl buffer at pH 8.0.

Kinetic parameters were obtained by fitting the experimental data to the Michaelis–Menten equation ($V_{0}=\frac{V_{max}[S]}{K_{m}+[S]}$) using nonlinear regression in GraphPad Prism version 8.0 for Windows (GraphPad Software Inc., San Diego, CA, USA). Protein concentration was quantified using the standard Bio-Rad protein assay (Bio-Rad, Hercules, CA, USA), with bovine serum albumin as the standard.

### 4.10. Effect of Surfactants on Esterase Activity

The enzymatic activity of the recombinant esterases was evaluated in the presence of different surfactants, such as sodium dodecyl sulfate (SDS), lithium dodecyl sulfate (LDS), Tween 80, Triton X-10, and cetyltrimethylammonium bromide (CTAB), at two concentrations, 1 and 10% *v*/*v*, in the enzymatic reaction mixture.

To assess their potential as detergent additives, the activity of the enzymes was also tested in the presence of commercially available liquid detergents such as Perla, OMO, Ariel, Quix and Sun. These detergents were added to the reaction mixture at final concentrations of 1% and 10% (*v*/*v*). Prior to enzyme addition, detergent solutions were heated at 80 °C for 1 h to inactivate the endogenous enzymes present in the commercial formulations, thus preventing any interference with the assay. The lipolytic activity was measured as described in Section 4.6.

### 4.11. Effect of NaCl on Esterase Activity

The effect of NaCl on the enzymatic activity was evaluated by incorporating different molar concentrations of NaCl (0, 0.5, 1.0, 1.5, 2.0, 2.5, 3.0, 3.5, and 4.0) into 50 mM Tris HCl buffer, pH 8.0. The relative activity of each of the enzymes was determined under these conditions. The lipolytic activity was measured as described in Section 4.6.

### 4.12. Effect of Inhibitors on Esterase Activity

Four enzymatic inhibitors, β-mercaptoethanol, PMSF, DTT, and EDTA, were evaluated for their inhibitory effects on the activity of Est1-ET, Ces1-ET and Plp1-ET at final concentration of 1 mM, 5 mM and 10 mM. The enzyme was incubated with each inhibitor prior to conducting the activity assay, and residual activity was then measured. The lipolytic activity was quantified as described in Section 4.6.

### 4.13. Effect of Divalent Cations on Esterase Activity

The enzymatic activity of Ces1-ET, Est1-ET and Plp1-ET under study was measured in the presence of different divalent ions. The effect of divalent cations on the activity of Ces1-ET, Est1-ET and Plp1-ET was evaluated by adding the following salts (CaCl_2_, MgCl_2_, MgSO_4_, ZnSO_4_, CuSO_4_ and BaCl_2_) at concentrations of 2 mM, 5 mM and 10 mM into a 50 mM Tris HCl buffer, pH 8.0. The lipolytic activity was measured as described in Section 4.6.

## 5. Conclusions

This study highlights *T. thermophilus* strain ET-1 as a valuable source of thermostable enzymes with potential industrial applications, demonstrating the diversity of thermophilic microorganisms present in Andean geothermal ecosystems in Northern Chile. The newly developed pTGT-1 vector allows the flexible expression of thermostable enzymes in both *E. coli* and the thermophilic host *T. thermophilus* HB27, facilitating the production and characterization of thermozymes of biotechnological interest in both hosts.

The characterized esterases (Ces1-ET, Est1-ET and Plp1-ET) exhibit high optimal temperature, alkaline tolerance, compatibility, and resistance to salinity, positioning them as potential candidates for industrial and biotechnology applications requiring thermozymes.

The *T. thermophilus* strain ET-1, and other extremophiles from the understudied El Tatio Geothermal Field, represent a promising reservoir of novel biomolecules and biotechnological innovations, supported by the unique microbial diversity that flourishes in these high-altitude Andean geothermal environments.

## 6. Patents

The data presented in this publication are associated with the international application published under the Patent Cooperation Treaty (PCT), Number WO 2023/184047, with a publication date of 5 October 2023. This application includes a biological material deposit made at Chilean Collection of Microbial Genetic Resources (https://www.cchrgm.cl/; accessed on 15 October 2021—RGM Cod. 3133). The patent was filed by the Universidad de Antofagasta and recognizes the intellectual property rights of the four authors of this publication affiliated with Universidad de Antofagasta.

## Figures and Tables

**Table 1:** Closest amino acids and nucleotide sequences to Ces1-ET, Est1-ET and Plp1-ET.

	Genus	Specie	Identity	GenBank Number	Functional Classification
Ces1-ET (amino acid)	*Thermus*	*thermophilus*	97.8%	WP_014629056.1	Alpha/beta fold hydrolase
	*Thermus*	*thermophilus*	97.3%	WP_244340854.1	Alpha/beta fold hydrolase
	*Thermus*	*thermophilus*	97.3%	WP_201350924.1	Alpha/beta fold hydrolase
Ces1-ET (nucleotide)	*Thermus*	*thermophilus TTHBAR1*	98.4%	LR027517.1	Thermostable monoacylglycerol lipase
	*Thermus*	*thermophilus JL-18*	98.4%	CP003252.1	Esterase/Lipase
	*Thermus*	*thermophilus SNM1-7*	97.8%	AP025603.1	Carboxylesterase
Est1-ET (amino acid)	*Thermus*	*thermophilus*	94.9%	WP_223965571.1	Pectin acetylesterase
	*Thermus*	*thermophilus*	94.6%	WP_165738509.1	Pectin acetylesterase
	*Uncultured bacterium*		94.6%	QRD81008.1	Lipolytic protein
Est1-ET (nucleotide)	*Thermus*	*thermophilus AK1-1*	98.0%	AP024937.1	Hypothetical protein
	*Thermus*	*thermophilus AA1-1*	98.0%	AP024926.1	Hypothetical protein
	*Thermus*	*thermophilus AA2-29*	97.8%	AP019794.1	Hypothetical protein
Plp1-ET (amino acid)	*Thermus*	*thermophilus*	87.2%	WP_143586050.1	Patatin-like phospholipase
	*Uncultured bacterium*		87.2%	QRD81043.1	Lipolytic protein
	*Thermus*	*thermophilus*	87.6%	WP_024119695.1	Patatin-like phospholipase
Plp1-ET (nucleotide)	*Thermus*	*thermophilus HB27*	90.2%	CP053287.1	Patatin-like phospholipase
	*Thermus*	*thermophilus HC11*	90.0%	AP019801.1	Phospholipase
	*Thermus*	*thermophilus AA2-29*	90.1%	AP019794.1	Phospholipase

**Table 2:** Sequence oligos used for esterases’ amplification.

Name	Primer Sequence
Ces1-FP (EcoRI)	GAATTCTATGCACCTTCTCCTCCTC
Ces1-RP (NotI)	GCGGCCGCTTGCAAGTAGTTATCACTTGC
Ces1-Rp (NdeI)	CATATGTTGCAAGTAGTTATCACTTGC
Est1-FP (EcoRI)	GAATTCTATGAAGCGCTTATCGCGCTGGT
Est1-RP (NotI)	GCGGCCGCAGGCCGCACCCGGGGGGG
Est1-RP (NdeI)	CATATGAGGCCGCACCCGGGGGGG
Plp1-FP (EcoRI)	GAATTCTATGCGCGGCCTCGTGCTT
Plp1-RP (NotI)	GCGGCCGCTCATACCTCCCCCACTCTACT
Plp1-RP (NdeI)	CATATGTCATACCTCCCCCACTCTACT

**Table 3:** Kinetic properties of Ces1-ET, Est1-ET and Plp1-ET.

Enzyme	*V_max_* (*µ*moles *p*-Nitrophenol/10 min)	*K_m_* (mM)
Ces1-ET	15 ± 4	0.25 ±0.05
Est1-ET	13 ± 2	0.20 ±0.01
Plp1-ET	18 ± 4	0.30 ± 0.05

The kinetic values correspond to the mean of three independent experiments. Reactions were carried out at 55 °C in 50 mM Tris-HCl, pH 8.0 using *p*NP-butyrate as the substrate and 1 µg of protein in the assay.

**Table 4:** Effect of metal ions, inhibitors, and detergents on the lipolytic activity of Ces1-ET, Est1-ET and Plp1-ET esterases.

	Relative Activity (%)					
	Ces1-ET	Est1-ET	Plp1-ET	Ces1-ET	Est1-ET	Plp1-ET
**Divalent Cations**	**2 mM**			**10 mM**		
CaCl_2_	100 ± 9	135 ± 6	105 ± 16	90 ± 14	110 ± 9	90 ± 27
MgCl_2_	90 ± 11	100 ± 20	105± 14	76 ± 27	93 ± 11	79 ± 20
MgSO_4_	98 ± 1	104 ± 1	93 ± 5	83 ± 14	59 ± 22	73 ± 22
ZnSO_4_	108 ± 1	138 ± 0	118 ± 0	80 ± 9	91 ± 10	96 ± 11
CuSO_4_	80 ± 5	77 ± 5	90 ± 0	65 ± 13	45 ± 15	73 ± 16
BaCl_2_	111 ± 14	134 ± 10	120 ± 10	73 ± 2	61 ± 8	59 ± 8
**Inhibitors**	**1 mM**			**10 mM**		
β-mercaptoethanol	35 ± 17	69 ± 22	55± 19	0 ± 19	0 ± 18	0 ± 17
phenylmethylsulfonyl fluoride (PMSF)	0 ± 7	0 ± 8	0 ± 3	0 ± 11	0 ± 2	0 ± 13
Ditiotreitol (DTT)	0 ± 15	0 ± 14	0 ± 19	0 ± 18	0 ± 29	0 ± 17
Ethylenediaminetetraacetic acid (EDTA)	22 ± 17	49 ± 15	30 ± 19	35 ± 19	79 ± 17	40 ± 18
**Surfactants**	**Concentration (1%)**			**Concentration (10%)**		
Sodium dodecyl sulfate (SDS)	84 ± 2	32 ± 8	82 ± 2	21 ± 11	14 ± 12	21 ± 12
Lithium dodecyl sulfate (LDS)	0 ± 1	26 ± 2	0 ± 2	0 ± 2	6 ± 2	0 ± 3
Tween 80	12 ± 24	142 ± 2	14 ± 26	0 ± 16	134 ± 3	0 ± 15
Triton x-100	0 ± 3	165 ± 1	0 ± 0	0 ± 9	166 ± 2	0 ± 0
Cetyltrimethylammonium Bromide (CTAB)	64 ± 6	29 ± 7	82 ± 7	18 ± 9	17 ± 11	21 ± 10
**Commercial Detergent**	**Concentration (1%)**			**Concentration (10%)**		
Perla	45 ± 8	102 ± 2	41 ± 9	35 ± 23	27 ± 10	34 ± 38
OMO	152 ± 3	81 ± 5	130 ± 3	43 ± 22	26 ± 20	40 ± 23
Ariel	181 ± 1	89 ± 2	150 ± 5	0 ± 5	21 ± 2	10 ± 1
Quix	97 ± 9	130 ± 3	90 ± 9	0 ± 16	16 ± 4	0 ± 16
Sun	99 ± 5	123 ± 3	80 ± 5	87 ± 7	15 ± 11	50 ± 18

The data are presented as mean ± standard deviation (SD, *n* = 3).

**Table 5:** Effect of NaCl in lipolytic activity.

[NaCl] (M)	Relative Activity (%)		
	Ces1-ET	Est1-ET	Plp1-ET
0	100 ± 0	100 ± 0	100 ± 1
0.5	76 ± 1	85 ± 1	89 ± 1
1.0	76 ± 7	80 ± 8	86 ± 9
1.5	55 ± 4	57 ± 2	66 ± 4
2.0	55 ± 3	52 ± 1	60 ± 7
2.5	50 ± 9	52 ± 1	56 ± 1
3.0	44 ± 7	45 ± 0	50 ± 1
3.5	40 ± 2	45 ± 1	49 ± 6
4.0	39 ± 1	41 ± 2	45 ± 3

The data are presented as mean ± standard deviation (SD, *n* = 3).

**Figure A7:**
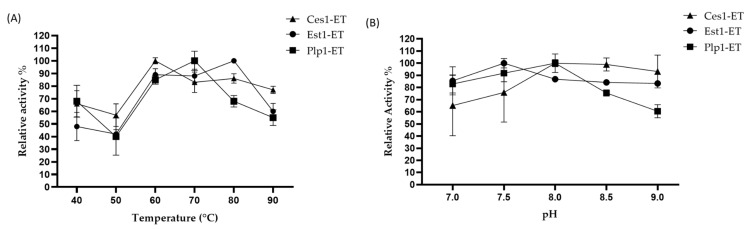
Properties of Ces1-ET, Est1-ET and Plp1-ET expressed in *T. thermophilus*. (**A**) Effect of reaction temperature on enzymatic activity in range 40–90 °C. (**B**) Effect of pH on enzyme activity using 50 mM Tris-HCl buffer across pH of 7.0–9.0.

## Data Availability

The sequence data for the characterized enzymes and the assembled genome of *Thermus thermophilus* strain ET-1 have been deposited in the NCBI database. Raw data from all experiments, including sequencing datasets, will be made available through an institutional server, in line with Universidad de Antofagasta’s commitment to Open Science, and will be linked to the DOI of the present publication. Full public access to all datasets will be granted once the patent process has been concluded, as early release of certain data could compromise intellectual property protection related to the esterases described in this study.

## Appendix A

**Table A1:** Accession numbers of the nucleotide and protein sequences described in the study.

*Thermus thermophilus* Strain ET-1	Acc. No. PRJNA1134705	
Esterase Name	Nucleotide Sequence Acc. No.	Protein Sequence Acc. No.
Ces1-ET	PV146438	XPQ45698.1
Est1-ET	PV146437	XPQ45697.1
Plp1-ET	PV146439	XPQ45699.1

**Table A2:** (Data Figure 3B): Enzymatic activity assay against two substrates, *p*NP-butyrate- and *p*NP-palmitate, considering Abs 410 nm/µg protein.

Abs 410 nm/µg Protein					
Ces1-ET	Est1-ET	Plp1-ET	Ces1-ET	Est1-ET	Plp1-ET
***p*NP-butyrate**			***p*NP-palmitate**		
0.139 ± 0.007	0.236 ± 0.030	0.134 ± 0.004	0.004 ± 0.001	0.024 ± 0.003	0.008 ± 0.001

The data are presented as mean ± standard deviation (SD, *n* = 3).

**Table A3:** (Data Figure 4): Properties of Ces1-ET, Est1-ET and Plp1-ET expressed in *E. coli*.

Relative Activity (%)			
**Optimal Temperature**			
**°C**	**Ces1-ET**	**Est1-ET**	**Plp1-ET**
40	60 ± 20	60 ± 12	56 ± 10
50	82 ± 6	74 ± 3	76 ± 9
60	100 ± 10	79 ± 3	93 ± 15
70	86 ± 7	96 ± 10	100 ± 18
80	40 ± 2	100 ±8	52 ± 31
90	17 ± 20	52 ± 7	14 ± 30
**Optimal pH**			
**pH**	**Ces1-ET**	**Est1-ET**	**Plp1-ET**
7	76 ± 5	93 ± 24	23 ± 8
7.5	100 ± 1	76 ± 18	54 ± 23
8	67 ± 3	70 ± 6	100 ± 4
8.5	83 ± 7	72 ± 4	69 ± 5
9	14 ±14	100 ± 15	28 ± 7

The data are presented as mean ± standard deviation (SD, *n* = 3).
